# Professional and Personal Physical Therapist Development through Service Learning in Collaboration with a Prisoner Reinsertion Program: A Qualitative Study

**DOI:** 10.3390/ijerph17249311

**Published:** 2020-12-12

**Authors:** Isabel Rodríguez-Costa, Ma Dolores González-Rivera, Catherine Ortega, Joana-Marina Llabrés-Mateu, María Blanco-Morales, Vanesa Abuín-Porras, Belén Díaz-Pulido

**Affiliations:** 1 Nursing and Physiotherapy Department, Alcala University, 28805 Alcalá de Henares, Spain; isabel.rodriguezc@uah.es (I.R.-C.); belen.diazp@uah.es (B.D.-P.); 2 Biomedical Sciences Department, Alcala University, 28805 Alcalá de Henares, Spain; marilin.gonzalez@uah.es; 3 Physical Therapy Department, Health Science Center, San Antonio, Texas University, San Antonio, TX 78229, USA; ortegac2@uthscsa.edu; 4 Consellería d’Educació, Universitat I Recerca del Govern Illes Balears, 07009 Palma, Spain; joanamarinallabres@gmail.com; 5 Faculty of Sport Science, Universidad Europea de Madrid, 28670 Madrid, Spain; maria.blanco@universidadeuropea.es

**Keywords:** development, physical therapy student, prisoners, service learning

## Abstract

There is a great concern whether Physical Therapy students upon completion of their educational program are ready and equipped with the requisite skills to construct and implement a successful patient intervention with culturally diverse groups. The purpose of this study is to describe the professional and personal physical therapist development of Physical Therapy students after participating in Solidarity Activities in Collaboration with a Prisoner reinsertion program as a service-learning course. A qualitative approach was used. A convenience sample of twenty physical therapy students doing service learning and one teaching professor were included. Student diaries were analyzed. Semi-structured interviews were done to explore five students’ and the professor’s judgements. Internal and external observations and filling out structure field-notes were also used as data triangulation in order to build the conceptual model. The main findings include that the application of knowledge and practice of skills in different environments are the most important skills attained with this service learning. Five key themes emerged from the data analysis, namely: application of knowledge, adaptation to different environments, improving communication with patients, assisting people and providing treatment with self-confidence. A recommendation is that Physical Therapy programs include workplace practice in different environments to enhance the development of professionalism among students.

## 1. Introduction

Healthcare professionals need to be prepared to confront a challenging world and provide quality patient interventions. Educational formation is the first step to attaining this goal. The need to develop personal and professional skills is well-recognized as enabling future physical therapists to function efficiently [1]. Nowadays, managing stress and enhancing resilience are important considerations for becoming successful in physical therapy interventions [2].

To prepare students for the interactive world in multicultural environments it is important to practice in varied environments [3]. One of the special groups that physiotherapists treat, and which students could practice with, is the prisoner population. This is the group of interest that would participate in a reinsertion program. Because it is vital for this community to learn healthy physical exercise habits, the prisoner environment is for physiotherapists a new emerging workplace. In this context, students can apply their theoretical knowledge [4] and practice their communication skills [5]. Physical therapists could expand their profession, because professions evolve as a result of their interrelations with others [6]. The prison location is another place where a physical therapist could be considered a primary care practitioner [7].

In this framework emerges “Solidarity Activities in Collaboration with a Prisoner Reinsertion Program” (SACP) as a service-learning course in a Spanish Physical Therapy program. This course was created with the objective of giving physical therapy care to special groups who in other conditions would not have access to this care [8]. One of these under-served groups are prisoners who participate in a community reinsertion program thorough sports, called “Running Makes You Free.” Within the course, physical therapy students create and deliver educational sessions to inmates once a month. The information presented includes different physical therapy techniques and healthy sports habits. This course was created to provide students extra clinical practice in varied environments [9].

Physical therapy education requires applied learning as a central part of the formation. This kind of learning includes learning skills in the workplace [10]. It is known that experiential learning is the best manner to develop clinical reasoning expertise [11]. Experiential learning facilitates socialization in a practice community, it is collaborative work [12] and gives the opportunities for students to apply theoretical knowledge, to integrate it into practical settings and to build their own learning process [13,14,15]. It is a form of interactive learning and was added to this curriculum to enhance educational development as well as translational learning for this group of physical therapy students [16].

In situ experience for students outside the university facilitates personal grown, active engagement and promotes motivation and interest for learning [17,18]. Furthermore, this kind of experiential learning encourages physical therapists to be lifelong learners [19] and to adapt their knowledge to changing care demands [20,21,22,23].

In light of the evidence, it seems important to create and facilitate in situ experience outside the university for Physical Therapy students to develop clinical expertise [24,25] because it promotes situated learning [26,27,28]. In addition, this type of learning can enhance career development [29,30,31]. Experiential practice education promotes main areas of the physical therapists’ formation such as: application of skills to the clinical context [32,33] improved confidence and clinical reasoning [34,35].

The aim of this study is to describe the professional and personal development of Physiotherapy Program students after participating in SACP as a service-learning course in their educational curriculum deepens undergraduates’ and teachers’ perceptions.

## 2. Materials and Methods

### 2.1. Design

Qualitative research methods with a grounded theory approach were used [36]. This study reports on students’ and professors’ perceptions during and after participating in SACP in the Grade of Physical Therapy [37,38]. Data collection was concurrent with the study reported and this contributed to an inductive research process [24].

Twenty students who had been doing the SACP and one professor who teaches these activities were identified for participation in the study. Purposive sampling was used to select five men and fifteen women students and teacher from SACP of 2011–2012 course. The mean age of the students was 22.6 years (SD ± 2.81, range 21–29). The professor had ten years of experience teaching at university and five years’ experience teaching SACP. Purposive sampling identified the participants to ensure they were typical of the phenomenon being studied [39,40]. All participants, twenty students and the professor, voluntarily agreed to participate and gave informed consent.

### 2.2. Data Collection

Four data collection tools were used. Each student completed a written diary after each session. There were eight sessions along the academic course and ten students participated in each educational session, so eighty written student diaries were collected.

Face-to-face audio-recorded semi-structured interviews were done (Table 1). Five motivated students and a professional teacher of SACP were interview two times. Because one of the SACP teachers was a main researcher, there was a prior relationship between the researcher and students and the professional teacher, so that the participants felt comfortable and could be honest about their reflections and responses. The first round of interviews took place before SACP started in the academic course. The second round of interviews was done after their participation in SACP. A total of twelve face-to-face interviews (ten with the students and two with the teacher) were recorded.

Internal and external observations were done [41]. The professor and a physical therapy teacher who does not teach in SACP filled out a structured field note after each educational session so that sixteen structured field notes were compiled.

Data from diaries, interviews, as well as internal and external observations were triangulated. The knowledge gleaned over four years of SACP was considered data as observational memory to contextualize, and contributed to the data analysis. Ethical approval was gained from the Research Unit of the Penitentiary Center. The project was approved by the Spanish General Secretariat of Penitentiary Institutions review board 1632-20/07/2012.

### 2.3. Data Analysis

Initially, the process of analysis involved transcripts of the structured field notes and students´ diaries. The first data analysis step was data immersion. The thematic analysis was guided by the systematic approach of Grounded Theory [42]. The analysis was done through a series of coding: open, axial and selective, and the constant comparative method [42] contributing to the creating conceptual and analytical framework. This analysis approach was used to try to explain the personal and professional development in the Physical Therapy program through the SACP.

Data were managed and organized using Atlas.ti, version 5.2 (ATLAS.ti Scientific Software Development GmbH, Berlin, Germany), a qualitative software package version. This software facilitated the interactive process of grounded theory [43]. Interview passages, field notes and diary quotations were coded and subjected to comparison and differentiation. Similar concepts were clustered to form categories [44]. Final coding involved the interrelation categories and theory building thorough a network. The conceptual framework is presented and explained in the findings.

### 2.4. Trustworthiness

Participants’ bias was controlled by an honest and trusting research–participant relationship that was established over a period of three years [45]. The researchers and participants had a shared knowledge of the SACP and therefore, this could be considered as insider research [46]. The absence of researcher bias was ensured by a constant verification and triangulation of the data from students’ diaries, students’ interviews, the professor’s interviews, and the internal and external observations.

## 3. Results

Data from students’ diaries, students’ interviews, professor’s interviews, internal and external observations were analyzed and showed good agreement with most components of the constructs and order of importance (Table 2). Students’, teachers’ and external observations supported the importance of applying knowledge and creating workplace practice.

**Figure 1:**
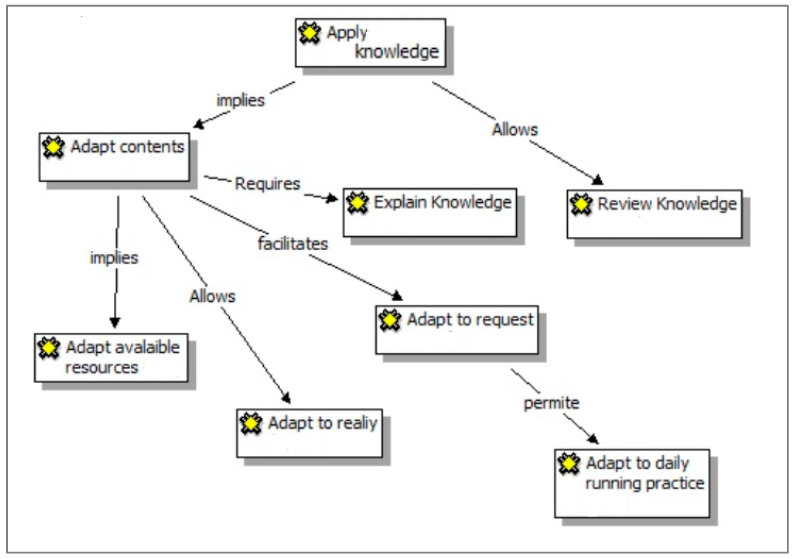
Create workplace practices.

Five categories of professional and personal physical therapist development were generated from the data. The text and information in Figure 1 explain how the concepts are linked and describe their properties and dimensions according to the conceptual density required in grounded theory studies.

Findings include quotations from participants presented in italics with names omitted to ensure participants’ confidentiality. To provide trustworthiness, quotations are included to collect participants’ experience. Quotations are referenced with a code that identifies its place within the analyzed document; for example, ISI1, 24 means that the quote is from the Initial Student Interview of student 1 and is in the 24th min of the audio.

### 3.1. Applying Knowledge

A primary finding, supported by previous literature, was the importance of applying knowledge [13,14]. Several concepts are related to this category: reviewing knowledge and adapting knowledge to reality and available resources. Some students appreciate the satisfaction after having the opportunity of applying their learned knowledge, as cited below (Figure 1):

“The opportunity of a lot of things, review, deepen and apply to practice, a lot of things.”

(ISI3, 3)

“It is something that you have to review to apply, understand it and explain it, and this helps you to make this more automatic.”

(FSI1, 9)

Furthermore, the teacher emphasizes that student participation in the SACP provides the opportunity to review, apply and adapt knowledge:

“I think that it is very interesting that an unexpected event in the practice with prisoners appears, because it is a good opportunity for students to learn and adapt them-selves to changes.”

(SFN1, 88)

“It forces them to make an effort to adapt something that they know to concrete situations and in that situation they have to create rather than be given each step.”

(FTI1, 15)

### 3.2. Create Workplace Practise

The second identified theme was the importance of creating workplace practice for students. This category is related to other concepts such as appreciating multicultural differences, treating different people, open-mindedness, interest and situated learning.

“All the mates have gained in flying hours, in experience, in being able to face new things.”

(FSI4, 15)

“I noticed what the physical therapist reality outside the classroom is, because beyond the techniques there are other situations that we have to manage.”

(FSI2, 19)

“You have a more open mind to different situations and people that are in some kind of inequality.”

(ISI5, 2)

“This practice allows us to know a little of the unknown reality beyond our comfortable lifestyle.”

(SD4, 22)

Creating workplace practice was considered to be an essential element of the educational development for students. Students and teachers recognize the important relationship between procedural knowledge needed for treating patients and theoretical knowledge gained during the formal university process, therefore, they relished the opportunity to treat patients in a workplace practice setting such as collaborating with a prisoner reinsertion program with a special population.

“For students, it is a big help to practice with people for their future professional development.”

(SFN1, 28)

“Students can treat a minority group in a reinsertion centre with physiotherapy sport techniques.”

(ITI1, 10)

“It is a big help to face real patients.”

(SD5, 38)

### 3.3. Communication

Communication is another central theme found in the results. Communication with the patient, with the family and with the team is a necessary skill for an effective physical therapy intervention. Communication is a bidirectional process; the physical therapist needs to gain information and also to provide information to those involved in the patient’s intervention [47].

“In this kind of sessions, we can improve our confidence and consolidate our knowledge, not only in an academic level in the physiotherapy framework but in facts such as expression and communication.”

(SD6, 402)

For physical therapists, it is important to be able to interpret the lay speech of the patient and then to give adapted explanations to the patients’ understanding [47]. Students have been able to experience the importance of a fluent communication with the patient to meet the patient’s particular and special needs as a prisoner in a reinsertion program.

“We do not only make things relate to physiotherapy, but also with personal communication. Besides you lose all your prejudices.”

(SD6, 234)

“Physical contact, between students and inmates in physiotherapy techniques is really positive to facilitate communication.”

(SFN2, 7)

### 3.4. Helping People

Assisting people is a final theme that emerges from the data. It is not a primary finding but it is at the end of the entire process. Because this is the ultimate result, this helps students to feel satisfaction and they feel themselves being a useful person.

“You feel yourself useful; you help people that in other circumstances would not have this help, my expectations have been overcome.”

(FSI1, 34)

“Solidarity Activities allow students to see another kind of reality, to become more sensitive, to understand their profession as a way to help others; this is for me the biggest satisfaction.”

(FTI1, 2)

“It is very rewarding to see how your ideas and knowledge learnt during years is beneficial for the others.”

(SD6, 44)

“We have helped people, to improve their health and their quality of life.”

(SD5, 40)

### 3.5. Professional Skill

The last category found was the importance of developing professional skill as an important aspect of being an efficient professional. Professional skill, also called expertise or ability, is a quality that has to be attained. At the time that students start their career and participate in this type of service learning, they gain extra practical time.

“Some students have pleasantly surprised me; because I have seen really good management with the group.”

(FTI1, 26)

“Students have to manage uncertainty and to make decisions to adapt themselves to the reality.”

(SFN1-88)

Skill is closely related to self-confidence and security. Participants enhance the opportunity thorough this SACP to develop their skill in a real workplace practice with a special population.

“You improve your confidence, you manage better with them. I have seen a progression in me.”

(FSI5, 2)

“We have successfully managed in a new situation for us.”

(SD9, 60)

“The session has been very rewarding as much on a professional level as a personal level and it has helped me enhance my self-confidence.”

(SD8, 248)

## 4. Discussion

It is well known that the use of workplace practice in different contexts and environments provides more active teaching and learning methodologies [48]. Based on Bandura’s Social Learning Theory [49], modelling examples allows students to learn by observing a model perform a task and then learn by doing. Several studies include the importance of learning from examples adapted to the prior students’ experience [50,51]. For novice students, instructions are more effective than problem solving; for advanced students, it is the opposite, because they have prior knowledge of the task and have acquired a schema for solving a problem [52]. Therefore, advanced students benefit from practice.

Experiential learning can be organized in widely different environments. This can contribute to physical therapy students’ development. One essential skill for the healthcare professional is the understanding of culturally diverse classes of people [53,54]. Cultural competence is the combination of cultural awareness, cultural knowledge and cultural sensitivity [55]. Physical therapists need to practice with culturally diverse groups to develop this competence and therefore, educational curricula for physical therapists should offer this opportunity. Working with inmates has been a good initiative to work on cultural sensitivity and has helped students to lose their prejudices.

The five themes found supports previous evidence about professional and personal development of physical therapy students. The findings complement other recent research on translational learning and clinical expertise in Physical Therapy [4,56,57].

Themes and resulting theory were generated from data derived directly from students’ and teachers’ experience. Research findings suggest that SACP intervention promotes the application of knowledge and the creation of workplace practice that facilitates student development as lifelong learners and also improves motivation for learning. These results are similar to findings from several previous studies [17,18].

Taking part in the course SACP during educational studies helps students to experience a challenge. It is important for them to face this challenge, manage it and solve problems that arise in the situation. As other studies have shown [17,18,42], it is important to include the workplace practice in professional education courses as a central aim for students’ education. In this workplace practice, a real-life situation is when students really have to use all their skills to achieve a successful patient intervention.

Managing uncertainly also enhances students’ self-confidence and facilitates clinical reasoning. As Davis and Burnand’s (1992) research found, to generate doubt is closely related to spiral learning, in which a student can return to another level of practice or study. Knowledge and experience are not simply piled but are integrated and promote a much deeper and specialized level of knowledge and skills [58].

In contrast with previous studies [59], in which students doubt their ability to apply theory to practice, it was found with this research that there is good agreement between this term of ‘ability’ and its importance for physical therapy students’ development.

On the other hand, no agreement was found between the students’ ability to adapt their communication level to the patients’ characteristics [57]. In contrast with previous studies regarding postgraduate students, where a high level of communication with the patients was discovered, in this study a discrepancy was found. While students feel an improvement in their communicative skills, teachers and external observation do not agree and, in actuality, they think that students need even more workplace practice with special populations to develop this skill.

More attention has to be focused on reflecting on the understanding of the importance of communication and new methodological strategies could be introduced in the next courses to pay more attention to the development of this particular skill, with specific practice activities. In addition, this discrepancy could have occurred because this intervention was done with undergraduate students and the research cited was with postgraduate and, therefore, older, more experienced students.

Furthermore, the results support that students who take this course improved their special technical skills, as was found in previous studies [24,25,33]. This finding demonstrates an improvement in clinical expertise development after completing SACP, related with the opportunity for the students to gain practice and apply adapted knowledge in different environments and populations.

Finally, there was a high level of satisfaction [60] and motivation among student participants after helping people with special characteristics and needs. All participants expressed deep happiness after taking SACP. This, in fact, is good for their mental health and facilitates interest and motivation among students [60,61], two main characteristics of lifelong learners [62].

### Limitations

It is important to note that the final theory of professional and personal physical therapist development through SACP was developed from students’ and teachers’ perceptions. A further limitation was that it was carried out only with students of SACP. The research results were obtained from data co-created with participants, and as such are caught in a specific time and place. Although findings cannot be automatically generalized, they could be transferrable to other similar situations.

## 5. Conclusions

The research results suggest that the intervention, namely SACP, promotes the application of knowledge, the creation of workplace practice, and promotes the development of physical therapy students as lifelong learners, and improves motivation for learning. The findings of this study add to the growing body of evidence about the importance of situated learning and knowledge application in real clinical contexts. The participants gained in improving communication and enhancing their own self-confidence. The students increased their interest in learning and attained a deep feeling of satisfaction.

As such, these findings could provide some insight into meeting the needs of Physical Therapy education to promote students’ professional and personal development. It would be appropriate to continue this work further by exploring similar situations to enable analytic generalization.

### Lessons for Practice

One of the special workplace settings where physical therapy students could develop their practical and communicative skills with culturally diverse classes of people is a prison. This kind of experiential learning prepares students to construct and implement a successful patient intervention in different realities.

Although it is difficult to create the same workplace practice with a challenging population such as prisoners, it would be recommended for Physical Therapy degree programs to include workplace practice in different environments and with different populations to facilitate physical therapist development.

## Figures and Tables

**Table 1:** Interview agenda.

Interview	Date	Interview Agenda
Initial Interview	January 2012	Initial expectation; initial motivation; initial program knowledge
Final Interview	June–July 2012	Achieve of expectation; final experience; final learning

**Table 2:** Triangulation of data.

Component of the Construct	Students’ Diaries	Students’ Interviews	Professor’ Interview	Internal Observation	External Observation
Apply Knowledge	X	X	X	X	X
Workplace Practice	X	X	X	X	X
Communication	X	X	X	X	No adapted
Assist People	X	X	X	X	X
Professional Skill	X	X	X	X	X
